# Polysaccharides from the Marine Environment with Pharmacological, Cosmeceutical and Nutraceutical Potential

**DOI:** 10.3390/molecules21050551

**Published:** 2016-04-27

**Authors:** Nadia Ruocco, Susan Costantini, Stefano Guariniello, Maria Costantini

**Affiliations:** 1 Department of Biology and Evolution of Marine Organisms, Stazione Zoologica Anton Dohrn, Villa Comunale, 80121 Napoli, Italy; nadia.ruocco@szn.it; 2 Department of Biology, University of Naples Federico II, Complesso Universitario di Monte Sant’Angelo, Via Cinthia, 80126 Napoli, Italy; 3 Bio-Organic Chemistry Unit, Institute of Biomolecular Chemistry-CNR, Via Campi Flegrei 34, Pozzuoli, 80078 Naples, Italy; 4 CROM, Istituto Nazionale Tumori “Fondazione G. Pascale”, IRCCS, 80131 Napoli, Italy; s.costantini@istitutotumori.na.it; 5 Dipartimento di Biochimica, Biofisica e Patologia Generale, Seconda Università degli Studi di Napoli, 80131 Napoli, Italy; ste_guar@hotmail.it

**Keywords:** cosmeceutics, marine polysaccharides, nutraceutics, pharmaceutics

## Abstract

Carbohydrates, also called saccharides, are molecules composed of carbon, hydrogen, and oxygen. They are the most abundant biomolecules and essential components of many natural products and have attracted the attention of researchers because of their numerous human health benefits. Among carbohydrates the polysaccharides represent some of the most abundant bioactive substances in marine organisms. In fact, many marine macro- and microorganisms are good resources of carbohydrates with diverse applications due to their biofunctional properties. By acting on cell proliferation and cycle, and by modulating different metabolic pathways, marine polysaccharides (including mainly chitin, chitosan, fucoidan, carrageenan and alginate) also have numerous pharmaceutical activities, such as antioxidative, antibacterial, antiviral, immuno-stimulatory, anticoagulant and anticancer effects. Moreover, these polysaccharides have many general beneficial effects for human health, and have therefore been developed into potential cosmeceuticals and nutraceuticals. In this review we describe current advances in the development of marine polysaccharides for nutraceutical, cosmeceutical and pharmacological applications. Research in this field is opening new doors for harnessing the potential of marine natural products.

## 1. Introduction

Marine species represent about one half of the global biodiversity, containing different and representative species and belonging to the main *taxa* also comprising a vast number of microbes and viruses. About 70% of the Earth’s surface is covered by the oceans, which correspond to about 90% of the biosphere and offer a great source of novel compounds. In the last decades, marine organisms have been extensively explored as potential sources of novel bioactive compounds [1]. During their evolution the different marine organisms such as bacteria, macro- and microalgae, sponges and fish have developed various kinds of defense mechanisms, based on the use of a great variety of specific and potent natural molecules, which enable them to survive a hostile environment that includes extreme conditions involving different degrees of salinity, pressure, temperature and light [2], as well as microbial and viral attacks.

Marine organisms thus represent a rich source for the discovery of novel natural compounds, comprising both small molecules (terpenoids, polyethers, polyketides, lipoproteins, and small antimicrobial peptides), usually used as defense systems against predators, and macromolecules with biotechnological potential, such as proteins, glycoproteins, and polysaccharides, that have also been identified. These molecules are not used in defense systems, but they have other important biological roles in marine organisms as cell surface receptors [3], in cell development and differentiation [4] and the innate immunity system [5]. They probably represent a very ancient defense system, reorganized during evolution [6,7], due to the direct contact of marine organisms with their environment, which has high concentrations of bacteria, pathogenic viruses and fungi. 

A great number of medicines or drugs have been isolated from terrestrial organisms, whereas far fewer medicine or drugs have been obtained from marine sources. This is in contrast with the high level of biodiversity in the marine environment, offering a great deal of opportunity for the discovery of marine natural products. This is mainly due to the fact the marine environment has not yet been as extensively explored as a potential source of potential medicines or drugs. Nevertheless, a variety of compounds has been obtained from marine organisms and are currently under study and in advanced stages of clinical trials. Some of them have already been marketed as drugs [8,9,10].

Among marine compounds, marine carbohydrates are considered important organic components of marine sediments [11,12]. In the biosphere, carbohydrates are the major organic compounds produced by photosynthetic organisms used as source of energy for heterotrophic organisms [13,14]. They are also important because of their participation in the immune system, fertilization, and food storage. Because carbohydrates are ubiquitous and abundant, they play an important role in biogeochemical cycles, occurring in the marine water column and sediment-water interface. In the marine system, total carbohydrates are present in monosaccharide, disaccharide, and polysaccharide forms [15,16,17] and are some of the most important organic compounds that are produced by photosynthesis in marine living organisms. 

Carbohydrates have received broad attention and are extensively studied by many investigators throughout the world [11,18,19,20,21]. A number of these studies have focused on the relationship between carbohydrates and organic carbon and on their distribution [22]. Of the different classes, polysaccharides have storage and structural roles both marine and terrestrial organisms. Glycogen and starch are storage polysaccharides, while the structural units are polysaccharides like cellulose and chitin. The storage forms of carbohydrates are unstable. They are utilized and degraded by *in situ* heterotrophic organisms while they deposit the organic matter from the surface to depths [23]. Besides the polysaccharides, monosaccharides are useful for humans and can cure many diseases, mainly those linked to metabolism deficiency such as diabetes [24]. 

Among carbohydrates, marine polysaccharides have various applications and people have used them for a long time due to their recognized human health benefits. Recently, much attention has been given to the structural and compositional properties of marine carbohydrates. Marine organisms, being very rich in carbohydrates, mostly in the form of sulfated and non-sulfated polysaccharides, represent a good resources of nutrients. A good example are carbohydrates extracted from marine algae, which have attracted the attention of several research groups because of their wide range of important biological activities with applications in the food, pharmaceutical and cosmetic industries. Seaweeds contain a significant amount of sulfated polysaccharides, used in the cosmeceutical and pharmaceutical industries. Seaweed-derived sulfated polysaccharides also have potential uses for blood coagulation, antiviral activity, antioxidant activity, and anticancer activity. Many other marine organisms are also rich in polysaccharides, such as sulfated galactans. Furthermore, other marine polysaccharides, such as agar and alginates, have several applications in food production and the cosmeceutical industry. For example, agar has been extensively used in medicinal or pharmaceutical industrial applications, as a suspending agent for radiological solutions (such as barium sulfate), as a bulk laxative with a smooth and non-irritating hydrated bulk in the digestive tract. It is also applied as an ingredient for tablets and capsules to carry and release drugs [25].

**Figure 1:**
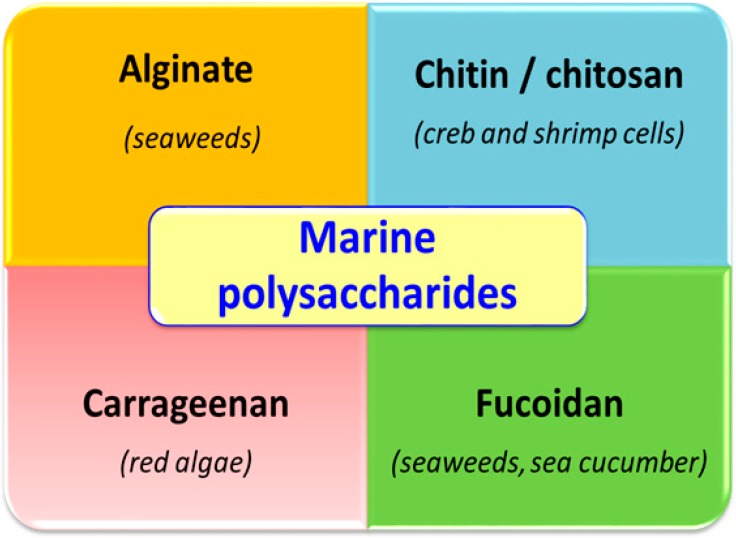
Marine polysaccharides of interest for cosmeceutical, nutraceutical and pharmacological applications. The marine organism sources are also reported.

In this review we describe the current advances in the use of marine polysaccharides (including chitin, chitosan, fucoidan, carrageenan and alginate; Figure 1) for nutraceutical, cosmeceutical and pharmacological applications.

## 2. Cosmeceutical Applications

**Figure 2:**
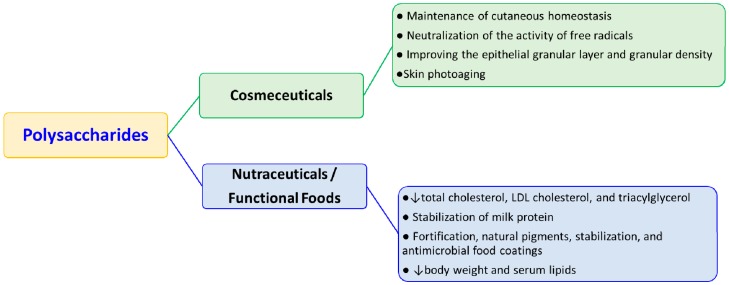
The cosmeceutical and nutraceutical applications of marine polysaccharides.

Cosmetics are products applied to the human body for its cleansing, beautification and appearance alteration without affecting its structure and functions. Some (such as sunscreens or antidandruff shampoos) can also used to prevent some diseases, concerning for example the structure of the human skin and in these cases they are also considered as drugs. Taking into account this consideration, Kligman introduced the term “cosmeceutical” about 20 years ago to define cosmetic products applied for personal care that have a combination of cosmetic and pharmaceutical uses [26,27,28,29]. Cosmeceuticals contain active ingredients delivered in the form of creams, lotions, and ointments and ingestible beauty products that are offered as liquids, pills and/or functional foods. They are formulated with ingredients or nutrients useful to promote healthy skin, hair and nails at the cellular level, including as key ingredients vitamins, minerals, botanical extracts and antioxidants (Figure 2).

Recently, great interest has been shown by consumers in novel bioactive compounds from marine natural sources, instead of synthetic ingredients, thanks to their perceived beneficial effects. Marine organisms have been demonstrated as rich sources of structurally diverse biologically active compounds with great cosmeceutical potential [30,31,32]. The increasing advances in marine biotechnology are offering great help in studies on aging, inflammation, and skin degradation linked to free radicals. At the same time, dermatological research suggests that the marine bioactive ingredients used in cosmeceuticals may have greater benefits beyond the traditional moisturizer role (e.g., [28]).

Structural polysaccharides that represent major constituents in plant and microbial cell walls and diverse marine organisms have been used in many types of industries. Chitin, for example, found in the structural backbone of the exoskeleton of crustaceans (crab and shrimp shells, forming crystalline structures to protect crustaceans from predators), and the exopolysaccharides (EPS), secreted by marine bacteria, offer greater potential in industrial applications. Chitin is a crystalline polysaccharide able to interact with several cell compounds in living human tissue. The first studies demonstrated that chitin nanofibrils maintain cutaneous homeostasis and neutralize the activity of free radicals, and represent a natural carrier for transcutaneous penetration of active principles. Ito *et al.* [33] verified the effect of chitin nanofibrils and nanocrystals on skin, using a three-dimensional skin cell culture. Their findings revealed that nanofibrils and nanocrystals can be applied in improving the epithelial layer and increasing of granular density of skin. In addition, chitin nanofibrils and nanocrystals application to the skin induced a lower production of TGF-β compared to that of the control group, thus suggesting skin protective effects. Chitin can be mechanically altered under acidic conditions to form chitin nanofibrils, which are biomaterials that are fully compatible with human skin cells, non-toxic and biodegradable. These nanofibrils are capable of forming complexes with other compounds, such as vitamins, carotenoids and collagen, facilitating their transcutaneous penetration [34]. Specific properties of these complexes including degree of cross-linking density, water content and dimension determine how readily actives are released and the depth of penetration into the skin.

On the other hand, chitosan (a linear polymer obtained by the partial deacetylation of chitin) is composed by polysaccharide chains of glucasamine and *N*-acetylglucosamine with free amino groups, interacting with other biological molecules. It is a cationic pH-sensitive polymer, which can be molded into various shapes including beads, hydrogels, nanofibers and nanoparticles. As a hydrogel, chitosan has superior water absorbing properties, making it valuable as a moisturizer. Chitosan oligomers stimulate fibroblast production, provide wound healing benefits, and exhibit antioxidant and metalloproteinase inhibiting effects. Another important attribute of chitosan is its broad antimicrobial activity that includes bacteria, yeast and fungi. Chitosan, in the form of nanoparticles, acts as a delivery system. These particles help to protect from environmental factors, such as light and oxidation, and facilitate their delivery to the skin. Thus chitosan has been identified by industry as a novel ingredient with multiple applications in cosmeceutical formulations [27,35].

Among marine exopolysaccharides, an EPS secreted by *Alteromonas macleodii* has already found application in cosmetics [36,37]. Other different polysaccharides, including fucoidan, carrageenan, alginate and agar, have been used as texture-improving agents in the cosmeceutical industries for their beneficial cosmetic effects. In fact, the cell walls of marine algae are rich in various bioactive polysaccharides: fucoidans in brown algae, carrageenans in red algae and ulvans in green algae. Fucoidan from various brown seaweed sources (*Saccharina japonica*, *Fucus vesiculosus*, *Undaria pinnatifida* and *Hizikia fusiformis*) and marine invertebrates, such as sea cucumber [30], represents the most abundant polysaccharide and the most commercially available. It is a highly sulfated polysaccharide, made up primarily of l-fucose, exhibiting diverse biological activities [34]. In recent years, fucoidans have been investigated to develop novel cosmetic products thanks to their property of reacting with the surface of the skin forming a protective layer that enhances skin hydration [34], when applied topically to the skin.

In skin-related diseases, UV-B reduces type I procollagen levels, increased matrix metalloproteinase-1 (MMP-1) levels in human skin and plays a major role in the photoaging process [38]. Furthermore, fucoidan treatment increased type I procollagen mRNA and protein expression in a dose-dependent manner, suggesting that it may prevent UVB-induced MMP-I expression and inhibit down-regulation of type I procollagen synthesis. According to these results, fucoidan has been suggested to have wide application as a potential agent to prevent and treat skin photoaging [39]. Considering that brown edible algae are rich in fucoidan and are a dietary foodstuff, their consumption could be beneficial in reducing the risk of MMP-related diseases [40]. Another research group reported the MMP inhibitory effect of a fucoidan fraction from seaweeds on the parameters involved in connective tissue breakdown. In more details, this *in vitro* study demonstrated that this fucoidan was able to successfully inhibit gelatinase with a secretion and stromelysin 1 induction by interleukin-1β on dermal fibroblasts. In addition, *ex vivo* studies have revealed that this polysaccharide was able to minimize human leukocyte elastase activity, to protect human skin elastic fibers against enzymatic proteolysis [41]. These findings clearly suggest the potential role of seaweed fucoidans in reducing the risk of some inflammatory pathologies involving extracellular matrix degradation by MMPs [42].

Carrageenan represents one of the most studied sulfated polysaccharides from marine red algae in the cosmeceutical field [30,43]. It is a sulfated galactan, composed of d-galactose units. Thanks to its physical and functional ability and antioxidant activity, carrageenan is an important product in the cosmetic and cosmeceutical industries, and is utilized for its antiaging, antioxidant, and anticarcinogenic activity (see below). The gelling ability of carrageenan is useful in producing a thicker texture with higher consistency in cosmetic production. In fact, many products such as skin lotions, toothpaste binders, and shaving foams utilize carrageenan isolated from marine algae [30,43].

Alginate is found in marine organisms cell walls, such as seaweeds. It is made of two units of guluronic and mannuronic acids, and is highly dependent on pH and temperature modification. The first alginate applications in the cosmeceutical field date back to 1927 [30]. Alginates have a wide range of applications in the cosmeceutical industry because of their high stability, thickening and gelling agent properties [30]. The biological activities of alginates are closely linked to the molecular weight, sulfate content and anionic groups, which give it antioxidant activity [44]. Alginate bioactivity depends on the presence of molecular weights of sulfated content and anionic group that makes antioxidant activity. For example, it is applicable in skin grafting in plastic surgery. In addition, it has applications in wound healing, because of hydrogel formation and degradability and providing a moist environment for wound [45].

## 3. Nutraceutical Applications

The term nutraceutical derives from joining the terms “nutrition” and “pharmaceutical”. It refers to foods or food ingredients with medical or health benefits. Through food-based approaches active substances with pharmaceutical properties are given to the humans to prevent or treat certain diseases linked to food. Several active compounds produced by different marine organisms have a wide role in the nutraceutical applications. These marine-derived active ingredients (including polyunsaturated fatty acids, polysaccharides, polyphenols, bioactive peptides and carotenoids) are known for their anticancer, anti-inflammatory, antioxidant, and antimicrobial activities and are applied as nutraceuticals, for example, to combat obesity [25,46,47,48] (Figure 2).

Human existence depends on meeting our basic physical necessities. The need to eat food is one of these necessities. In order to respond to this need, man has explored Nature to find foods, so for example, the history of fishing dates back 40,000 years. Due to the wide range of environments and organisms that survive underwater, biomolecules derived from marine organisms represent a large untapped reservoir of bioactive ingredients, often produced efficiently under unique conditions, such as low temperature or high pressure, that can be used in various food applications, to provide added nutritional benefits to foods and “natural” pigments, preservatives, or flavors [46,47]. Some important species of algae are of nutritional interest [49]: Phaeophyceae, brown algae *Ascophyllum nodosum*, *Ecklonia cava*, *Ecklonia kurome*, *Laminaria digitata*, *Lessonia flavicans*, *Saccharina japonica*, *Sargassum horneri*, *Undaria pinnatifida*; Cholorophyta, green algae, *Caulerpa racemosa*, *Codium fragile*, *Codium pugniforme*, *Gayralia oxysperma*, *Monostroma latissimum*, *Ulva australis*, *Ulva conglobata*, *Ulva lactuca*; Rhodophyta, red algae, *Cryptonemia crenulata*, *Grateloupia indica*, *Gigartina skottsbergii*, *Nemalion elminthoides*, *Nothogenia fastigiata*, *Pyropia haitanensis*, *Schizymenia binderi*. Most of these species have higher biomass and their possible edibility is an attractive characteristic feature for using them in medicinal foods by direct consumption through the diet and indirect consumption through their extracted nutraceuticals and functional food molecules [46]. The anticoagulant sulfated galactans and fucans from *Ulva fasciata*, for example, have uses in functional foods and nutraceuticals. Microalgal biomass rich in carbohydrates has been used directly for animal feed. Marine waste materials can in turn be redirected to process them for the extraction of carbohydrate molecules of nutraceutical interest [50]. Shellfish wastes from scallops (*Chlamys hastate*), cockles (*Cerastoderma edule*, *Clinocardium nuttalli*), whelks (*Buccinum undatum*), clams and mussels (*Mercenaria mercenaria*, *Mytilus galloprovincialis*, *Mytilus edulis*), oysters (*Crassostrea gryphoides*, *Crassostrea gigas*), and crustaceans (crab *Cancer pagurus*; lobster *Nephrops norvegicus* and *Homarus americanus*; shrimp *Crangon crangon*) have been redirected toward the development of various biopolymers, which can be used as nutritional substances and animal feed. With the emerging interest in using animal foods, especially of marine origin, the rate of consumption of several shellfish species has been increasing annually, although the processing of these shellfish wastes is still costly and only a few regions in the world would be able to produce the required quantities of chitin, chitosan, and their derivatives to meet the demand in the biological and biomedical fields [51].

Most Asian countries use macroalgae as foods for human consumption. Microalgae also have wide industrial applications, for example, as gelling, stabilizing and binding agents. The antioxidant properties of marine algal polysaccharides have been represented an important point in developing them as potential functional foods and nutraceuticals [49]. It is important to consider that the dietary fibers of seaweeds contain valuable nutritional substances. For these reasons, in the last years there has been increased attention paid to the use of seaweeds as functional foods for human consumption with nutraceutical and medicinal applications [52,53].

Reduced plasma total cholesterol, LDL cholesterol, and triacylglycerol (TAG) have been observed, attributable to the polysaccharides in edible seaweed [54]. Marine carbohydrates such as algins and exopolysaccharides from cyanobacteria can be used for the stabilization of emulsions or as bioflocculants. These properties allow a wide variety of unique food products to evolve. Polysaccharides are a common solution in food product formulation problems to achieve a certain texture, mouthfeel and body by thickening the food. Most polysaccharides have an ability, consisting in a viscosity increase or decrease with increasing shear rate, once they are dispersed in water. Some stabilizers result in a certain solution yield value, *i.e.*, a shear stress or applied force below which the solution will not flow (e.g., ketchup). Because of the thickening effect and the yield value, addition of suitable polysaccharides to an aqueous system can stabilize the suspending dispersed phase (could be a solid, liquid, or gas) and prevent it from separating out. Carrageenan has a unique functional property in its reactivity to proteins and for this property it is usually used to stabilize milk protein [55]. Normally, carrageenan is used in combination with other hydrocolloids such as starch, locust bean gum, and carboxymethyl cellulose. Furcellaran has a similar function, is used but less extensively in food. Many functional requirements and various applications such as fortification, natural pigments, stabilization, and antimicrobial food coatings are met by the use of simple and complex carbohydrates derived from marine foods. Cyanobacteria from marine environments also represent an important source of exopolysaccharides: for example, *Cyanothece* sp. *ATCC 51142* produces a polymer capable of gel formation very useful in food industries [56].

Red algae like *Gelidium*, *Gracilaria*, *Hypnea* and *Gigartina* are the main sources of agar [57,58]. Agar E406 has been used in the food industry for gel formation and food gums, as well as food additives, thanks to its properties as an emulsifying and gelling agent [59,60].

Recently, chitooligosaccharide (COS) has been studied in the nutraceutical field for its antidiabetic [61] and hypocholesterolemic [62] properties and adipogenesis inhibition [63]. In the food industry, chitosan and COS have been used as dietary food additives [64] and as dietary supplements to decrease body weight and serum lipids [48]. The importance of the application of chitosan depends from the fact that: (i) it is not specifically digested in the gastrointestinal tract by binding and precipitating fat in the intestines, so that it is not absorbed; (ii) has the property to swell to give a feeling of satiety in the stomach; (iii) is able to reduce the absorption of dietary fat in intestines through inhibition of pancreatic lipase activity. The cationic chitosan can link to the fatty acids and bile acids, interfering with emulsification of neutral lipids like cholesterol and other sterols by binding them with hydrophobic interaction, thus reducing intestinal absorption of fat and cholesterol.

## 4. Pharmacological Applications

**Figure 3:**
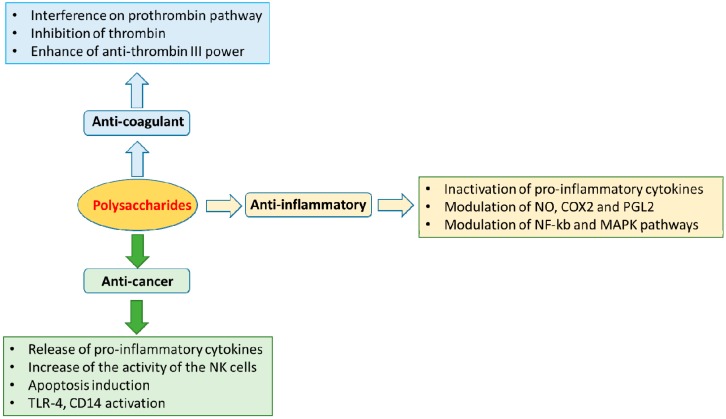
Properties of marine polysaccharides and their applications in pharmacology.

The resurgence of natural products-initiated drug discovery is tied to the exploration of novel natural resources and organisms, such as those in the marine world, which represents the largest unexplored resource. In the past decade a dramatic increase in the number of preclinical anticancer lead compounds from diverse marine life form sintering human trials has been reported. It is also important to consider that Nature is been considered an ancient pharmacy. New trends in drug discovery from natural sources emphasize investigation of the marine ecosystem to explore its numerous complex and novel chemical entities. These entities are sources of new leads for treatment of many diseases such as cancer, AIDS, inflammatory conditions and a large variety of viral, bacterial and fungal diseases [65]. Among natural products from the marine environment, the marine carbohydrates represent a good challenge in pharmaceutical field, because of their anti-inflammatory, immunomodulation, anti-coagulant and anticancer activities (Figure 3). Another application of polysaccharides in pharmaceutical industries consists of, for example, the use of agar and agarose beads for sustained release of water soluble drugs [66]. This application is based on the significantly lower sulfate content of these two compounds, on their better optical clarity and increased gel strength. For example, water soluble and hypnotic drugs have been prepared with agar beads (instantaneously form by gelification), containing phenobarbitone sodium. These studies indicated that agar beads can be used for the preparation of sustained release dosage forms.

### 4.1. Anti-Inflammatory and Immunomodulatory Activities

In “*in vivo*” studies the heterofucan from the seaweed *Dictyota menstrualis* (Phaeophyceae, brown algae) induced an inhibition of leukocyte migration with a related decrease in the levels of pro-inflammatory cytokines [67]. On the other hand, the fucoidan from the alga *E. cava* decreased cyclooxygenase-2, nitric oxide and prostaglandin E2 levels [68,69]. The polysaccharides from the green seaweed *Ulva rigida* [70] and the marine dinoflagellate *Gymnodinium impudicum* [71] activate the production of nitric oxide and immunostimulate the production of cytokines in macrophages.

Other molecules from *U. pinnatifida* (Phaeophyceae), *Porphyridium* (Rhodophyta), *Phaeodactylum* (Bacillariophyta), and *Chlorella stigmatophora* (Chlorophyta)had immune-suppressant effects in “*in vitro*” and “*in vivo*” studies by blocking the Th2 activity [72,73].

However, Tabarsa *et al.* [74] reported that the polysaccharide from *Codium fragile* (Chlorophyta) was able to induce NO release only when it was bound to the protein moiety by activating NF-κB and MAPK pathways. These authors demonstrated that in the case of polysaccharides from *C. fragile* their sulfate content was not necessary for their activity [74] in contrast to the results published from Leiro *et al.* [70] on polysaccharides from the cyanobacterium *U. rigida*. In addition, some algal polysaccharides are able to bind toll-like receptor-4 or pattern recognition receptors, involved in the innate immune response [75,76].

Concerning chitin and chitosan, it has been shown that administration of chitin through the vascular system enhances the release of cytokines by macrophages [77]. Moreover, it up-regulates Th1 immunity and down-regulates Th2 immunity [78]. *In vivo* studies demonstrated that chitinase enzymes can increase immunity in the presence of infection. In fact, clinical trials were conducted on allergic and asthmatic patients, in which there is an over-expression of chitinases [79,80]. Moreover, considering the polymeric properties of the chitin, some authors have focused on the utility of chitosan polymer composites cross-linked with resin and demonstrated that they can be used as alternative vehicles for oral delivery of aceclofenac, a non-steroidal anti-inflammatory drug [81]. Also the chitosan (Ch)/poly-(γ-glutamic acid) (γ-PGA) nanoparticles created as vehicle for diclofenac, another non-steroid anti-inflammatory drug, resulted able to inhibit the prostaglandin E2 production of activated macrophages, and to stifle local inflammatory reactions [82].

Laminaran (or laminarin) was first discovered in the *Laminaria* species (brown algae), being the food reserve of all these algae. Laminaran is a water-soluble polysaccharide and has a good inhibitory effect on virus proliferation. It is able to inhibit the adsorption of HIV on lymphocytes and the activity of HIV reverse transcriptase. These results suggests that laminaran exerts a good inhibitory effect on HIV replication [83,84].

### 4.2. Anti-Coagulant Activities

In the literature it is reported that some carbohydrates from seaweeds have anticoagulant effects by inhibiting thrombin or by activating anti-thrombin III or by increasing the clotting time both in the intrinsic and extrinsic pathways. Moreover, these molecules can also have an antithrombotic activity by blocking thrombin activity, mediated through the heparin cofactor II [85,86,87,88,89]. However, other authors evidenced that they also interfere in the PT (prothrombin) pathway, and, therefore, are not able to affect the extrinsic coagulation pathway [90].

Furthermore, an important role of the content in sulfate has been assigned in the anticoagulant activities, as the presence of sulfate and its distribution pattern play an important role in the processes of coagulation and/or platelet aggregation [91,92]. In particular, in the case of some fucoidans and fucans, the anticoagulant properties resulted to be related: (i) to the content of sulfate or disulfate or fucose [91]; (ii) to the higher molecular weight that usually induced a stronger anticoagulant activity [93]; and (iii) if the molecule presents a linear backbone [94]. Laminaran is also an example of a marine polysaccharide which exerts anticoagulant activity after structural modification like sulfation, reduction or oxidation [95].

Some *in vivo* studies also showed the anticoagulant properties of marine carbohydrates by increasing the clot formation time [96]. A *S*-galactofucan from the brown seaweed *Spatoglossum schröederi* showed a strong antithrombotic activity in an *in vivo* study [97]. Moreover, spirulan from *Arthrospira platensis* interfered with the blood coagulation-fibrinolytic system and exhibited anti-thrombogenic properties [98].

The degree of sulfation of chitosan is an important point. In fact, highly sulfated chitosans induce an increase of thrombin, activated partial thromboplastin time and thrombin time [99].

### 4.3. Anti-Cancer Effects

As also reported above, chitin is certainly the most abundant biopolymer in the marine environment and it can be converted into chitosan, the acid-soluble form of chitin, by *N*-deacetylation. Some pharmaceutical functions of chitin and chitosan are due to their unique physicochemical properties. In fact, both are non-toxic, renewable and biodegradable. Chitin and chitosan are two polymers that represent promising therapeutic candidates with therapeutic applications in drug delivery and gene therapy [100]. Recently, chitosan samples obtained through enzymatic deproteinization of chitin from Norway lobster (*Nephrops norvegicus*) were used to evaluate their anti-proliferative capacity. In details, the cytotoxic effects of chitosan samples were tested on human colon carcinoma cells HCT116. Chitosan showed an anti-proliferative capacity against this cancer cell line in a manner dependent on the dose and the degree of acetylation [101].

Moreover, Muanprasat *et al.* [102] investigated the effect of COS on AMP-activated protein kinase (AMPK) in intestinal epithelial cells. COS activated AMPK in two human colorectal adenocarcinoma cell lines, HT-29 and Caco-2, and inhibited NF-κB transcriptional activity and NF-κB-mediated inflammatory response. Moreover, the oral administration of COS was able to block the development of aberrant crypt foci in a mouse model of colitis-associated colorectal cancer (CRC) by β-catenin suppression and caspase-3 activation [102]. Different carrier systems based on chitosan are prepared to study the release and the cellular permeability of different molecules and drugs. For example, naringenin encapsulated in nanoparticles had a release of about 5% in gastric fluid and cytotoxic effects on lung cancer cells [103]. Moreover, other authors created nanoparticles of caffeic acid conjugated chitosan (ChitoCFA/CMD) and incorporated doxorubicin into them. Mouse colon carcinoma cell line, CT26, treated with doxorubicin-incorporated nanoparticles revealed strong fluorescence intensity while free doxorubicin revealed weak fluorescence intensity, indicating that doxorubicin-loaded ChitoCFA/CMD nanoparticles are a promising vehicle for anticancer drug targeting [104]. Finally, Lee *et al.* [104] showed that chitosan-coated nanoparticles containing curcumin caused a significant cell viability reduction on a human oral cancer cell line in a way dependent both from the concentration and the time and reduced the cytotoxicity to normal cells, when compared with the free drug [105].

Among the carbohydrates having anti-cancer activity it is important to mention also the role played by both macro- and microalgae. In fact, *S*-fucoidans from *Cladosiphon okamuranus* (Phaeophyceae) have shown anti-proliferative activity on myeloid cancer and leukaemia cell lines by inducing cell apoptosis [106,107]. Moreover, the fucoidan from *Saccharina gurjanovae* (Phaeophyceae) is able to inactivate the epidermal growth factor receptor (EGFR), an important player in cell transformation, differentiation and proliferation [108,109].

Other polysaccharides from *Sargassum* sp. and *Laminaria* (Phaeophyceae) showed anti-cancer activity on lung cancer and melanoma [110], and on colon and breast cancer cell lines [111,112]. Moreover, a fucoidan extracted from the marine brown alga *U. pinnatifida* has been found to induce osteoblastic cell differentiation by increasing the activity of alkaline phosphatase and the levels of osteocalcin, and to have positive effects on bone morphogenic protein-2 that is the most important factor for bone formation, remodeling and mineralization [113]. Also the alga *C. racemosa* (Chlorophyta) polysaccharide (CRP) showed anti-cancer activity. In fact, all its fractions induced inhibition of both melanoma cells and of hepatoma (H22) tumors transplanted in mice [114].

From a metabolic point of view, these compounds induce the release of pro-inflammatory cytokines, such as IL-2, IL-12 and INF-γ, increased activity of natural killer cells, Toll-like receptor-4, cluster of differentiation 14, and competent receptor 3 that in turn are able to induce the production of nitric oxide and apoptosis [115,116]. Considering these properties of *S*-fucoidans, they resulted able to protect damaged gastric mucosa [117], and to inhibit the activity of *Helicobacter pylori* on the stomach mucosa of Mongolian gerbils and to block the development of gastric cancer [118]. Recently, an unfractionated fucoidan from the alga *A. nodosum* showed apoptosis effects on human colon cancer cells (HCT116) by activation of caspases 3 and 9 and the PARP cleavage that induced an alteration of mitochondrial membrane permeability [119]. Moreover, *S*-laminaran resulted able to reduce metastasis formation by inhibition of heparanase that is known to be associated with the metastasis process [120]. Also, six glycosylated polyhydroxysteroids isolated from the starfish *Culcita novaeguineae* showed cytotoxic activities on hepatoma, melanoma, and epidermoid, prostate and breast cancer cell lines [121]. On the other hand laminaran has been tested on colon cancer cells, HT-29 and LOVO, and resulted to be involved in ErbB and IGF-IR signaling pathways [122,123] and to increase the intracellular level of ROS and Ca [124]. In details, in HT-29 cells it induced cell death in a dose-dependent manner, decreased MAPK and ERK phosphorylation, and inhibited the heregulin-stimulated phosphorylation of ErbB2 [122,123].

Some carbohydrates are reported as able to block carcinogenesis. For example, the polysaccharide DAEB, isolated from the green alga *Ulva intestinalis* (Chlorophyta) and composed from rhamnose, xylose, galactose, and glucose, was tested on mice. DAEB induced the secretion of TNF-alpha and nitric oxide, phagocytosis and the lymphocyte proliferation [125].

In the case of the anti-cancer activity, the degree of sulfation may also have an important role. In fact, fucoidan fractions from brown seaweeds are able to inhibit the leukaemia development but not that of sarcoma in mice [126].

## 5. Conclusions

The marine environment contains a number of micro- and macroorganisms, which have developed particular metabolic mechanisms for the biosynthesis of secondary metabolites with specific activities, useful for their survival. Functional materials from the marine environment include polyunsaturated fatty acids, polysaccharides, minerals, vitamins, antioxidants, enzymes, and bioactive peptides. All these biologically active compounds provide great human health benefits and represent an inexhaustible source of materials for the pharmaceutical, nutraceutical and cosmeceutical industries. The recent advances in molecular biology approaches, Next Generation Sequencing and methods to isolate and cultivate marine microorganisms have greatly contributed to the exploration of the marine environment biodiversity.

In conclusion, the challenges to use marine resources in different fields linked to human health is fully in-line with the Horizon 2020 strategic activity: “targeted approach towards specific activities focusing on “...exploration of the … biodiversity ... for ... helping us to understand for example how organisms that can withstand extremes of temperature and pressure and grow without light could be used to develop new industrial enzymes or pharmaceuticals...”.
